# A Male Child With Combined Glucose-6-Phosphate Dehydrogenase Deficiency and Hereditary Elliptocytosis: The First Case Reported From Saudi Arabia

**DOI:** 10.7759/cureus.97824

**Published:** 2025-11-26

**Authors:** Badriah G Alasmari, Fahad F Al Munajjim, Shady Wafa, Ayman Abualama, Lina Elzubair

**Affiliations:** 1 Pediatrics, Armed Forces Hospital Southern Region, Khamis Mushait, SAU; 2 Pediatric Medicine, Najran General Hospital, Najran, SAU; 3 Pathology, Armed Forces Hospital Southern Region, Khamis Mushait, SAU

**Keywords:** glucose-6-phosphate dehydrogenase (g6pd) deficiency, hereditary elliptocytosis (he), hereditary elliptocytosis (he) in combination with glucose-6-phosphate dehydrogenase (g6pd) deficiency, supportive treatment, whole exome sequencing (wes)

## Abstract

Glucose-6-phosphate dehydrogenase (G6PD) deficiency is the most common human enzyme defect, predisposing individuals to acute hemolytic anemia upon oxidative stress. Hereditary elliptocytosis (HE) is a distinct, typically autosomal dominant disorder caused by red blood cell (RBC) membrane skeleton defects, leading to chronic hemolysis. The co-occurrence of G6PD deficiency and HE is exceedingly rare, often resulting in more severe and complicated clinical presentations. Here we report the first case of a five-year-old Saudi male from a consanguineous marriage who presented in the neonatal period with G6PD deficiency and recurrent hemolytic crises, including a severe episode following circumcision. At age five, he exhibited persistent severe anemia (hemoglobin 5.8 g/dl), chronic hemolysis (reticulocyte count 14.4), and significant hepatosplenomegaly. Next-generation sequencing (NGS) provided a rapid and conclusive molecular diagnosis by identifying the genetic mutations for both disorders.

## Introduction

Glucose-6-phosphate dehydrogenase (G6PD) deficiency is globally recognized as the most common human enzyme defect, affecting nearly 330 million people worldwide [1]. This hereditary, X-linked genetic disorder primarily impacts red blood cells (RBCs); the lack of functional G6PD enzyme leaves RBCs vulnerable to damage from reactive oxygen species. While generally asymptomatic, affected individuals face the risk of acute, potentially life-threatening episodes of hemolytic anemia when exposed to specific oxidative triggers [2]. Hereditary elliptocytosis (HE) is a distinct group of inherited blood disorders characterized by the presence of abnormally shaped elliptical or oval RBCs (elliptocytes) in the peripheral blood. Estimated to affect one in 2000 to one in 4000 people globally, HE is typically inherited in an autosomal dominant pattern [3]. The fundamental defect lies in the structural proteins of the RBC membrane skeleton, compromising the cell's stability and flexibility. HE represents a spectrum of red cell membrane defects, ranging from clinically silent carriers to severe hemolytic anemia [4]. The co-occurrence of HE combined with G6PD deficiency is an extremely rare condition with very limited published reports [5]. This combination represents two distinct genetic blood disorders that can significantly increase the risk and severity of hemolytic anemia. Studies suggest that individuals with both conditions tend to have the lowest average age of diagnosis and the most pronounced anemia compared to those with only a single condition [5]. Given the potentially compounding nature of the hemolysis, establishing a definitive diagnosis requires a comprehensive method capable of evaluating both enzymatic and structural etiologies. Whole exome sequencing (WES) offers such a comprehensive approach, allowing for the simultaneous identification of both the established X-linked G6PD defect and the underlying autosomal variant in genes like SPTA1 or SPTB-information crucial for fully characterizing disease severity and guiding management decisions (e.g., splenectomy) [6].

Here, we report the first case of a male patient from the southern region of Saudi Arabia diagnosed with hereditary elliptocytosis in combination with glucose-6-phosphate dehydrogenase deficiency.

## Case presentation

**Figure 1:**
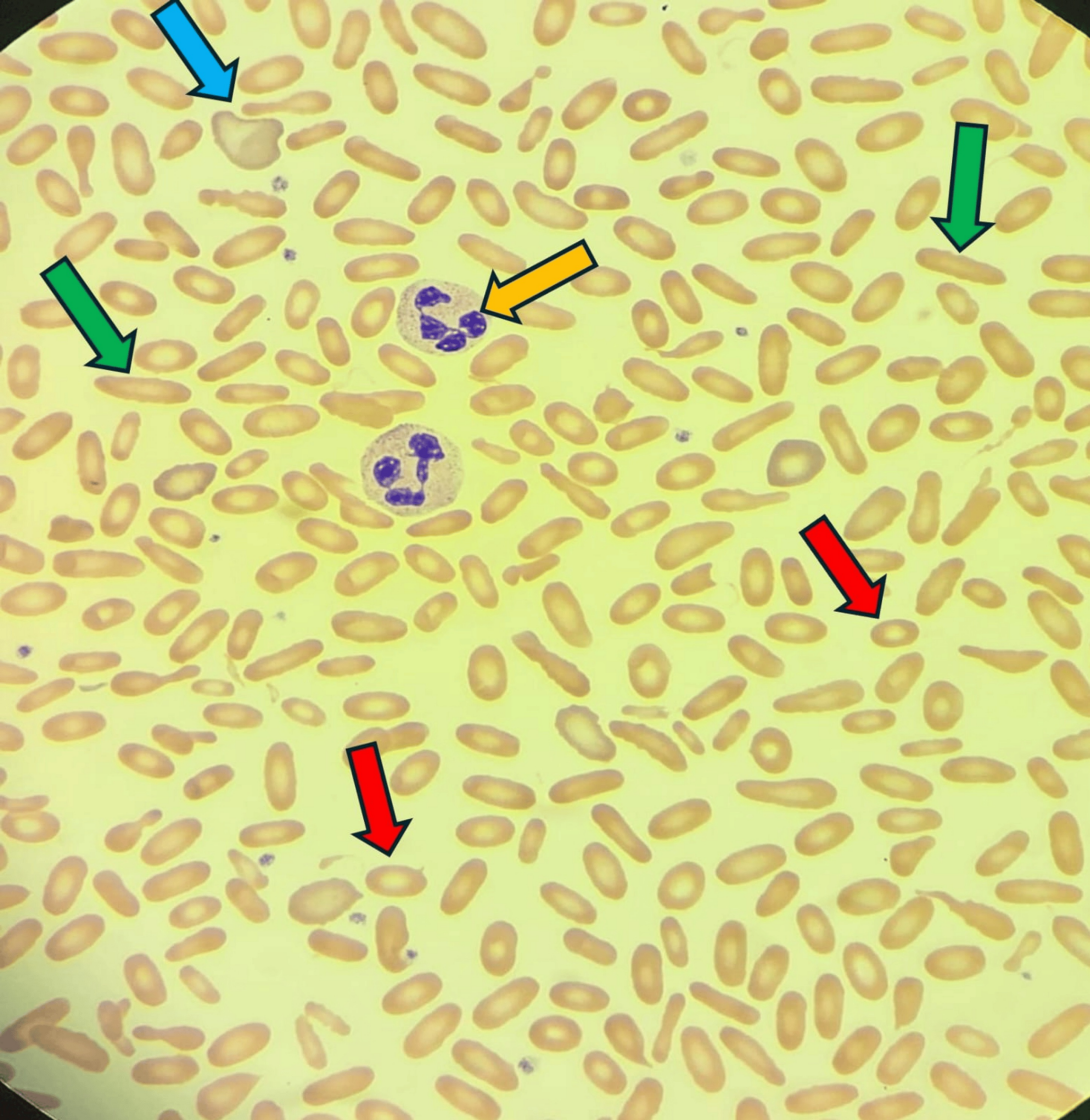
The peripheral blood smear demonstrates a high degree of poikilocytosis (variation in red blood cell (RBC) shape), which is a key feature in this case Elliptocytes (green and red arrows): The predominant feature is the presence of numerous elliptocytes (oval or rod-shaped RBCs). This is the hallmark finding of hereditary elliptocytosis (HE), confirming the morphological component of the patient's dual diagnosis. Pyropoikilocytosis/fragmented cells (red arrows): There are some irregularly shaped, fragmented, or teardrop-like cells, which are consistent with pyropoikilocytosis or general RBC fragility. The orange arrow points to a neutrophil. The blue arrow points to polychromatic cells.

**Figure 2:**
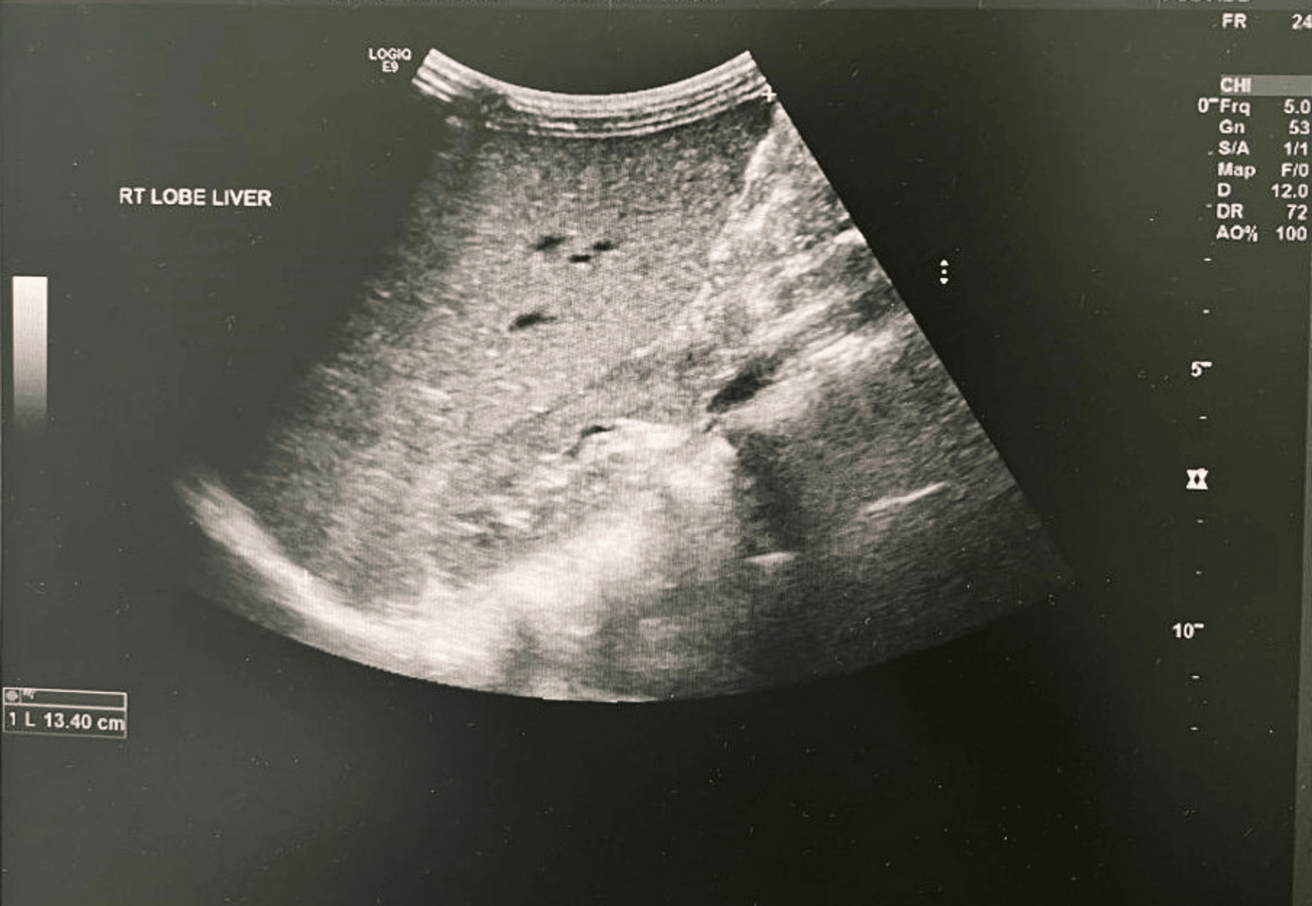
Ultrasound showing liver size 134mm

**Figure 3:**
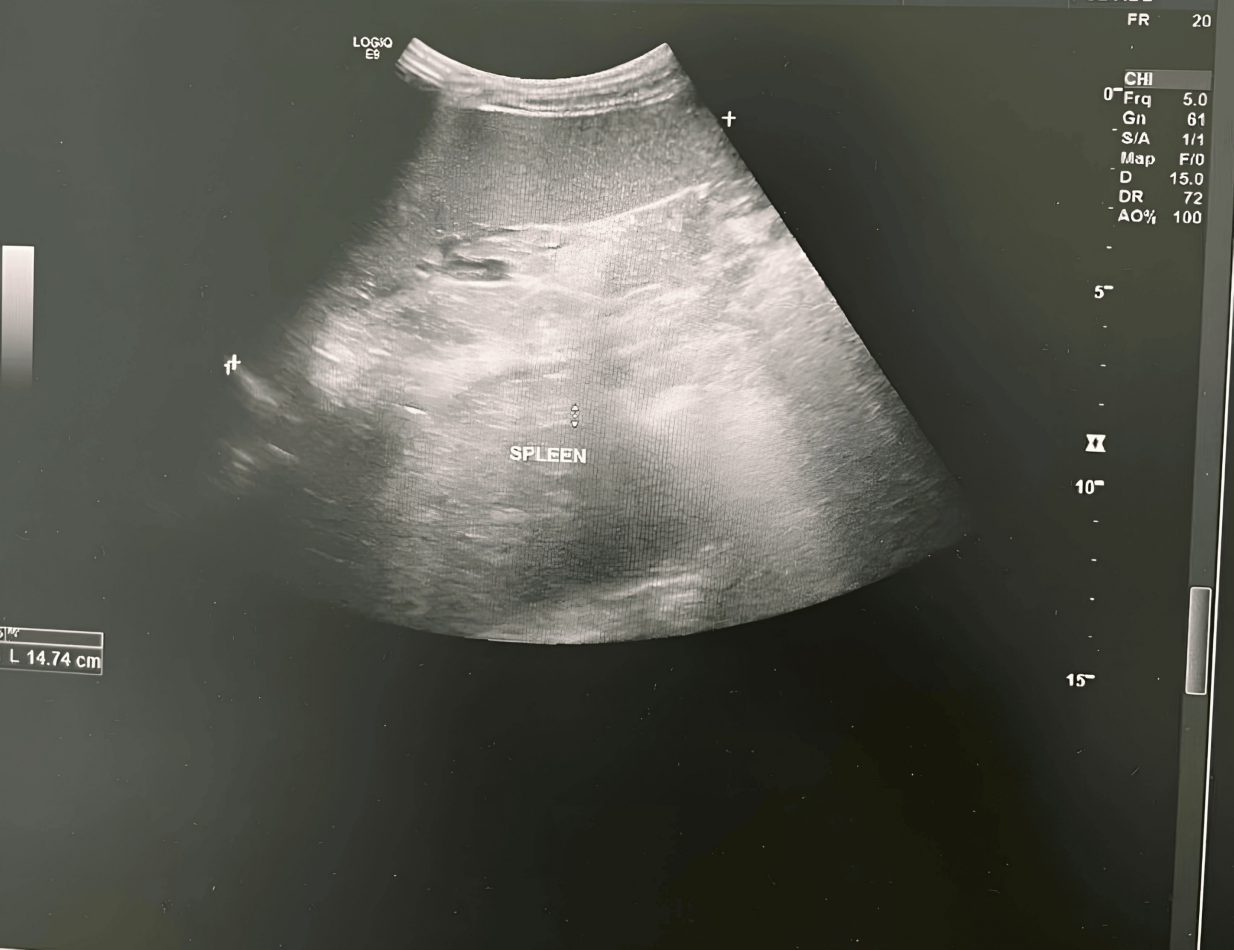
Ultrasound showing spleen size 147mm

A five-year-old Saudi boy from the southern region who was born after a full-term spontaneous vaginal delivery of a consanguineous marriage with a positive family history of his mother and both of his brothers with G6PD. Fifteen hours after delivery, the patient was noticed to be jaundiced with high serum bilirubin reaching 295 μmol/L, so the patient was admitted to the NICU with phototherapy. Both blood groups for the baby and the mother were O positive, the direct Coombs test (DCT) was negative, and the G6PD enzyme level came low at 4 U/g hemoglobin (HB) which is moderate deficiency. So the baby was admitted to the NICU for almost 14 days, then discharged, and the final diagnosis was G6PD, and the family was counselled about that and advised to follow up for further education. The patient was normal till the age of 50 days old, when he acquired pallor for four days following circumcision done at age of 43 days. The patient was admitted at that time, and the investigation came back with low HB at 6 g/dl and reticulocytes at 9.8%, with no organomegaly. So the patient received a blood transfusion for the first time in his life, and then after stabilization was discharged to home with a diagnosis of hemolytic crises of G6PD, and we gave him a follow-up appointment at our clinic for follow-up. Unfortunately the family did not attend their appointment for follow-up until he again came to our hospital at the age of five years with lethargy, pallor, and recurrent left upper quadrant pain. By examination there was hepatosplenomegaly, with the spleen measuring about 10 cm and the liver about 5 cm. HB was 5.8 g/dL with high reticulocytes at 14.4%, a G6PD enzyme level of 3.9 (moderate deficiency), and a negative DCT. The patient was admitted to our hospital for a blood transfusion for the second time in his life. After stabilization careful evaluation was done. All viral workup was negative, including cytomegalovirus and Epstein-Barr virus, elevated lactate dehydrogenase (LDH) level, slightly elevated serum ferritin level, and high indirect bilirubin level (Table 1). A peripheral blood smear was done and revealed moderate hypochromic microcytic anemia with pyropoikilocytosis and many elliptical-shaped RBCs, consistent with a diagnosis of HE (Figure 1). The ultrasound examination showed an enlarged liver around 134 mm (Figure 2) and a markedly enlarged spleen of about 147 mm (Figure 3) with no gallstones. So a WES study was sent for confirmation of the HE, and the result came back positive for a specific variant in the SPTA1 gene denoted as c.779T>C p.(Leu260pro). This variant is a homozygous pathogenic variant in the SPTA1 gene, and the results were consistent with an autosomal recessive SPTA1-related disorder. After confirmation of the diagnosis, the family was counseled about the patient's condition and to avoid triggering factors with the administration of folic acid as supportive treatment. 

**Table 1:** Provided critical laboratory evidence of a severe, uncompensated hemolytic state. The patient's profound anemia (5.8) g/dl was accompanied by definitive biochemical and hematological markers of active red blood cell (RBC) destruction, specifically a significantly elevated reticulocyte count, high LDH, and elevated indirect bilirubin. Crucially, the profile pointed toward the co-existing structural defect: the very high RDW is characteristic of significant variation in RBC size (anisocytosis). This finding, combined with the peripheral blood smear demonstrating pyropoikilocytosis and elliptocytes strongly supported the diagnosis of a membrane defect (hereditary elliptocytosis) superimposed on the known enzymatic deficiency of G6PD deficiency WBC = white blood cell count, HB = hemoglobin, PLT = platelet, HCT = haematocrit, MCV = mean corpuscular volume, RDW = red cell distribution width, LDH = lactate dehydrogenase, G6PD = glucose-6-phosphate dehydrogenase

Description	Result	Unit	Reference range
WBC	14.87	10^9^/L	4.5-13.5
RBC	2.62	10^12^/L	4.1-5.3
HB	5.8	g/dl	10.9-15
HCT	21.9	%	31-41
MCV	83.6	fl	73-98
RDW	25.6	%	
PLT	222	10^9^/L	150-450
Reticulocyte	14.4	%	0.5-2.5
LDH	483	mmol/L	155-290
Total bilirubin	44.5	μmol/L	< 34
Direct bilirubin	9.9	μmol/L	1.7-8.6
Ferritin	60	μg/l	10.3-55.8
HPLC (Hemoglobin Electrophoresis)			
HB A2	3.1	%	1-3.5
HB A	95.9	%	96.5-99
HB S	0	%	0
HB F	1	%	2

## Discussion

G6PD deficiency is recognized as the most common enzyme deficiency globally. As an X-linked hereditary disorder, it exhibits a higher prevalence in males; however, heterozygous females can also manifest clinical symptoms [1]. HE is a hereditary blood disorder observed across many parts of the world, including the Middle East. Notably, certain regions within the Middle East exhibit a high prevalence of genetic carriers, with rates exceeding 10% of the population carrying genes associated with hereditary blood disorders [7]. The co-inheritance of G6PD deficiency (an enzymopathy) and HE (a membranopathy) results in a compounding defect in the RBC, which generally worsens the severity of hemolysis compared to having either condition alone [8]. The combination of G6PD deficiency and HE creates an RBC that is simultaneously structurally weak and metabolically vulnerable. When both conditions are present, the clinical presentation is generally more severe than the relatively mild hemolysis typically seen in isolated HE or G6PD. The structural instability of the elliptocytic cell is combined with an impaired ability to neutralize reactive oxygen species. This synergistic effect means the dual-defective RBC is more likely to break down under normal conditions and is extremely susceptible to major hemolytic crises when exposed to G6PD triggers. While HE often causes mild chronic anemia, the addition of G6PD deficiency can lead to a more profound and persistent state of chronic non-spherocytic hemolytic anemia. The co-inheritance can complicate diagnosis. The characteristic elliptical shape of HE may still be visible on a blood smear, but the overall clinical picture of severe, chronic hemolysis can be misinterpreted as a severe variant of a single disease or another co-inherited condition (like thalassemia) [9].

In our case, the patient's recurrent hemolytic crises were attributed only to G6PD deficiency. This X-linked enzymopathy typically results in acute, episodic hemolysis following oxidative triggers but does not commonly lead to sustained splenomegaly. The development of a significantly enlarged spleen, therefore, strongly suggested an accompanying hemolytic disease that caused chronic red blood cell damage. This suspicion was confirmed when the peripheral blood smear showed distinct elliptocytosis, indicating a red blood cell membrane defect. This dual pathology - a structurally unstable cell (elliptocytosis) combined with a cell vulnerable to oxidative stress (G6PD deficiency) - explained the severity of the patient's symptoms. Ultimately, next-generation sequencing (NGS) provided a rapid and conclusive molecular diagnosis by identifying the genetic mutations for both disorders.

Management of G6PD and hereditary elliptocytosis remains supportive, focusing on transfusions when required, folic acid supplementation, and monitoring for iron overload. Splenectomy has been attempted in select patients, but with inconsistent outcomes. Genetic counseling is essential, especially in populations with high rates of consanguinity.

## Conclusions

This is the first reported case of combined G6PD deficiency and HE from the southern region of Saudi Arabia. This case highlights the role for advanced molecular diagnostics (WES) to fully characterize the etiology of severe or atypical hemolysis, which is crucial for guiding appropriate long-term management in these rare compound disorders. Clinicians must maintain a high index of suspicion for underlying structural defects when G6PD patients exhibit chronic, uncompensated hemolysis, as accurate dual diagnosis is crucial for appropriate risk stratification and guiding long-term management decisions, including the consideration of splenectomy. Management of this condition is still supportive with folic acid supplementation.
